# Long non-coding RNA HOTTIP enhances IL-6 expression to potentiate immune escape of ovarian cancer cells by upregulating the expression of PD-L1 in neutrophils

**DOI:** 10.1186/s13046-019-1394-6

**Published:** 2019-09-18

**Authors:** Anquan Shang, Weiwei Wang, Chenzheng Gu, Chen Chen, Bingjie Zeng, Yibao Yang, Ping Ji, Junjun Sun, Junlu Wu, Wenying Lu, Zujun Sun, Dong Li

**Affiliations:** 1 Department of Laboratory Medicine, Tongji Hospital of Tongji University School of Medicine, 389 Xincun Road, Shanghai, 200065 China; 2 Department of Pathology, The Sixth People’s Hospital of Yancheng City, Yancheng, 224001 China

**Keywords:** Ovarian cancer, Neutrophils, Interleukin-6, Programmed death-ligand 1, HOXA transcript at the distal tip, Immune escape

## Abstract

**Background:**

Long non-coding RNA (lncRNA) HOXA transcript at the distal tip (HOTTIP), has been demonstrated to be a vital biomarker when evaluating the prognosis of multiple cancers. Nevertheless, the potential function of HOTTIP in ovarian cancer (OC), a prevalent cancer among women worldwide, remains elusive. Hence, the current study aimed to elucidate the functional relevance of HOTTIP in the development of OC.

**Methods:**

Positive expression of PD-L1 and IL-6 was determined using immunohistochemical staining in the collected OC and normal tissues. The correlation of IL-6 and PD-L1 was analyzed using flow cytometry, Western blot analysis as well as Pearson’s correlation coefficient. The interaction among HOTTIP, c-jun and IL-6 was investigated with the use of RIP, ChIP and dual luciferase reporter gene assays. Finally, the effects of HOTTIP on T cell proliferation and infiltration were identified through gain- and loss-of-function studies in vitro and in vivo.

**Results:**

HOTTIP, IL-6 and PD-L1 were all highly expressed in OC tissues. A positive correlation was observed between IL-6 and PD-L1 and that between HOTTIP and IL-6 in OC tissues. HOTTIP was noted to promote the expression of IL-6 by binding to c-jun, which resulted in a promoted PD-L1 expression in neutrophils and immune escape while inhibiting T cell proliferation as well as tumor immunotherapy.

**Conclusion:**

Taken together, our study unveiled that HOTTIP could promote the secretion of IL-6, and consequently up-regulate the expression of PD-L1 in neutrophils, thus inhibiting the activity of T cells and ultimately accelerating immune escape of OC cells. Our study provides a potential therapeutic strategy by targeting HOTTIP in OC.

**Electronic supplementary material:**

The online version of this article (10.1186/s13046-019-1394-6) contains supplementary material, which is available to authorized users.

## Background

Ovarian cancer (OC) is considered as the most fatal gynecologic malignancy, and the commitments for early detection and novel therapies to lower its mortality have been principally unproductive [1]. Cancer immunotherapy has emerged as a promising therapeutic approach in oncology, and is characterized by the activation of immune system to induce tumor immune surveillance or to reverse the tumor immune escape [2]. Marth et al. have previously highlighted the promising therapeutic effect of immunotherapy in limiting OC [3]. In addition, therapeutic immunotherapy has also been demonstrated to have the ability to improve pancreatic cancer clinical trial outcomes [4]. However, some of the cases in the clinical trials failed as a result of “immune escape” [5]. The host immune system would respond to appearance of tumor cells, while neutrophils signify the first line of host defense against infection [6]. Besides, a previous study conducted on hepatocellular carcinoma (HCC) revealed that peri-tumoral neutrophils could negatively modulate the adaptive immunity through program death ligand 1 (PD-L1) and PD-1 pathway [7]. Regulatory T (Treg) cells are critical in immune tolerance in patients, and there is an inverse correlation between intertumoral Treg and patients’ survival [8]. Moreover, tumor-related neutrophils can recruit Treg cells, thereby promoting the development of HCC [9].

Long non-coding RNA (lncRNA) is a class of RNA molecules longer than 200 nucleotides that do not have functional protein-coding capacity [10]. The progression of OC has been suggested to be closely associated with lncRNAs [11]. For instance, the clinical significance of HOXA11 has been highlighted in predicting the prognosis of patients suffering from serous OC [12]. Moreover, HOXA transcript at the distal tip (HOTTIP) has been found to have a functional role in the pathogenesis of breast cancer, which is the most common cause of cancer-related death in women via regulation of one of its physical HOXA clusters, HOXA11 [13]. The results from the bioinformatics prediction revealed a biding site between HOTTIP and interleukin-6 (IL-6). IL-6 regulates chemokine generation and leukocyte apoptosis and is therefore a crucial checkpoint regulator of neutrophil trafficking in the inflammatory response [14]. IL-6 is known to have the ability to upregulate PD-L1 expression in tolerogenic antigen-presenting cells by elevating signal transducer and activator of transcription 3 (STAT3) [15]. PD-L1 suppresses host immunity through binding to its receptor PD-1 on lymphocytes, and promotes peritoneal dissemination, thus exacerbating OC [16]. Based on the aforementioned information, we proposed a hypothesis that HOTTIP may affect OC involving the regulation of IL-6 and PD-L1. Thus, the current study aims to investigate the mechanism by which HOTTIP/IL-6/PD-L1 axis influences immune escape of OC cells.

## Materials and methods

### Ethics statement

This study was conducted with the approval of the Ethics Committee of Tongji Hospital of Tongji University and The Sixth People’s Hospital of Yancheng City. Written informed consents were provided and signed by all participants prior to sample collection. All animal experiments were conducted in strict accordance with the recommendations in the Guide for the Care and Use of Laboratory Animals of the National Institutes of Health.

### Study subjects

A total of 53 cases of OC tissues were collected from patients with OC who underwent surgical resection at Tongji Hospital of Tongji University and The Sixth People’s Hospital of Yancheng City from May 2017 to October 2018. The average age of OC patients was (54.04 ± 11.88) years, out of which there were 32 patients > 50 years, and 21 patients ≤50 years. The clinical characteristics of the patients are listed in Table 1. In addition, normal fallopian tube fimbriae tissues were obtained from 25 patients with benign lesions near the uterus. The average age of normal subjects was (54.24 ± 10.57) years. A part of the specimen was used for the extraction of the total RNA and tumor-related neutrophils. The corresponding information of patients to the paraffin sections was recorded. Histologic diagnosis and grading were performed based on the standards of World Health Organization Classification of Tumors of the Digestive System. The clinical follow-up data included imaging examinations and assessment of survival of patients. The clinical tumor recurrence was defined as tumor recurrence or metastasis examined by clinical imaging. The overall survival (OS) was defined as the intermission between surgery and death.

**Table 1:** Clinical characteristics of the patients with OC

	Normal	Ovarian tumor
Case	25	53
Age (years)	54.24 ± 10.57	54.04 ± 11.88
> 50	16	32
≤ 50	9	21
Tumor stage (n)		
I		20
II		23
III		7
IV		3
Pathological classification (n)		
Serous		27
Mucinous		15
Endometrioid		9
Clear cell carcinoma		2

### Immunohistochemical staining

The sections were dewaxed with dimethylbenzene, hydrated with gradient alcohol, repaired in the citrate repair solution under high pressure and high temperature for 1.5 min, and cooled down at room temperature. After receiving a wash with phosphate-buffered saline (PBS), each section was incubated with 50 μL 3% H_2_O_2_ at room temperature for 20 min to eliminate the activity of endogenous peroxidase. Each section was then probed overnight with rabbit antibodies against PD-L1 (ab228415), IL-6 (ab9324) or CD3 (ab16669) in a 4 °C freezer, with the normal rabbit serum serving as the negative control (NC) instead. All of the above antibodies were purchased from Abcam Inc. (Cambridge, MA, USA). Next, each section was incubated with 50 μL polymer enhancer at 37 °C for 20 min and 50 μL enzyme-labeled goat anti-rabbit immunoglobulin G (IgG) at 37 °C for 30 min. Each section was added with 2 drops or 100 μL freshly prepared diaminobenzidine (DAB) for development and observed under a microscope for 3–10 min. A positive result was indicated by the presence of brown staining. After being washed with distilled water, the sections were counterstained with hematoxylin, dehydrated with gradient alcohol, mounted and observed under the microscope.

The expression of PD-L1 and IL-6 was characterized by the presence of fine brown granules in tumor cells. Grading was performed in accordance with the percentage of positive cells and the staining intensity: 0 point, the rate of positive cells ≤10%; 2 points, 11–51% positive cells; 3 points, 51–81% positive cells; 4 points: the rate of positive cells ≥81%; 1 point, weak intensity; 2 points, medium intensity; 3 points, high intensity. The total score was calculated by adding the two scores above: negative expression (−): positive cells ≤10 (irrespective of the staining intensity); weak positive expression (+): 3 points; positive expression (++): 4–5 points; strong positive expression (+++): 6–7 points.

### Collection and preservation of blood samples from patients

The patients with OC confirmed by pathologists after surgery were subjected to peripheral venous blood collection using anticoagulant tubes. The blood was then transferred to the laboratory in an immediate manner and stored at 4 °C for subsequent experiments.

### Cell culture

The 293 T cells were obtained from the Cell bank of the Chinese Academy of Sciences (Shanghai, China), and SKOV3, OVCAR3, and Hy-A8 cells were obtained from the American Type Culture Collection (ATCC, Manassas, VA, USA). These cell lines were cultured in Dulbecco’s modified Eagle’s medium (DMEM; 31,800,022, Gibco, Carlsbad, CA, USA) containing 1.5 g/L NaHCO_3,_ 10% fetal bovine serum (FBS) and 1% penicillin and streptomycin. Firstly, 5 mL medium was transferred into 15 mL sterile centrifuge tubes with sterile pipettes and water-bathed at 37 °C to melt the cells quickly. The cells then underwent centrifugation at 800 rpm for 5 min and were re-suspended in 1 mL culture solution. The cell suspension was subsequently cultured in 6 cm culture dish added with 2 mL solution in an incubator with 5% CO_2_ at 37 °C. The cell growth was observed the following day and the culture solution was changed every 1–2 days.

### Neutrophil isolation and identification

Extraction and identification of neutrophils from peripheral blood were conducted. Briefly, a total of 5 mL fresh heparin anticoagulant was collected from healthy adults. The serum was diluted with aseptic PBS at the ratio of 1:1. Afterwards, 3 mL of lymphocyte separation medium was added into a 15-mL centrifuge tube, followed by careful addition into the diluted serum. The tube was then centrifuged at 2000 rpm for 20 min. The intermediate layer of peripheral blood mononuclear cells (PBMCs) was transferred to a new centrifuge tube, washed twice with PBS and then centrifuged at 1500 rpm for 10 min. After resuspension with Roswell Park Memorial Institute (RPMI)-1640 medium containing penicillin and streptomycin, a flow cytometer was employed for the sorting of the CD66b^+^ cells (130–104-413, 1:10; Miltenyi biotec, Bergisch Gladbach, Germany).

Tumor infiltrated neutrophil separation was conducted. In short, the tissues were cut into 0.5 cm × 0.5 cm blocks, transferred into a tube with 10 mL DMEM medium, 100 μL collagenase IV, 100 μL DNase I, 100 μL MgCl_2_ as well as 50 μL CaCl_2_, and then mixed evenly. The tissue blocks were centrifuged and ground, and filtered with a 200-mesh steel mesh. The single cell suspension was harvested in a centrifuge tube (50 mL) and centrifugation was carried out for 10 min at 1700 rpm. The cells were re-suspended with PBS and filtered again. Next, the cells were collected in two centrifuge tubes (1.5 mL) and centrifuged for 5 min at 2000 rpm. Finally, the CD66b^+^ cells were sorted with the use of flow cytometry.

### T cell isolation and identification

Heparin anticoagulant was added and the cells centrifuged, after which the serum was discarded. The erythrocytes were lysed with 3 volumes of erythrocyte lysis buffer at room temperature for 10 min. After centrifugation for 5 min, the cells were washed with 5 mL PBS, centrifuged, and re-suspended with sterile PBS followed by cell counting. The CD3^+^ cells were sorted by flow cytometry with the PE-labelled CD3 antibody (130–113-129: 1:50; Miltenyi biotec, Bergisch Gladbach, Germany).

### Flow cytometric analysis of surface marker and intracellular proteins

The cells were dispersed into single cell suspension and re-suspended by the staining buffer (BD Biosciences, San Jose, CA, USA). The neutrophils were identified using FITC-labeled anti-CD66b (130–104-41,313, 1:10; Miltenyi-biotec, Bergisch Gladbach, Germany). Allophycocyanin (APC)-labeled PD-L1 antibody (130–117-694, 1:10; Miltenyi-biotec, Bergisch Gladbach, Germany) was used to determine PD-L1 expression. PE-labeled CD3 antibody (130–117-139, 1:50; Miltenyi-biotec, Bergisch Gladbach, Germany) was used for the separation of T cells and pacific blue-labeled interferon (IFN)-γ antibody (#505817, 1:50; BioLegend, San Diego, CA, USA) was used for the measurement of IFN-γ content. The detection of intracellular proteins required a membrane-breaking perforation of the cells. Flow cytometry was performed on the FACS Canto II analyzer (Becton Dickinson, Franklin Lakes, NJ) and the data were analyzed by the Flow Jo software.

### Cell treatment

The pLKO.1 plasmids (containing Puromycin resistance gene) knocking down HOTTIP, IL-6 or c-jun, the pBABE plasmids (containing Puromycin resistance gene) overexpressing HOTTIP or c-jun and the lentiviral package plasmids (pVSV-G, pREV, pMDL, PIK) were purchased from Cyagen (Suzhou, Jiangsu, China). The plasmids were transduced to the E.coli DH5α competent cells. The corresponding plasmids were extracted using plasmid extraction kit (DP103–03, TIANGEN Biotechnology Co., Ltd., Beijing, China). The plasmid was transduced into the HRK 293 T cells in strict accordance with the instructions of the Turbofect transfection reagent (R0531, Thermo Fisher Scientific Inc., Waltham, MA, USA). After 12 h, the culture medium was replaced, and collected at 24th and 48th h, respectively. The viruses were obtained after being filtered with the 0.45 μm filter, after which the OC cell line was infected. The corresponding cell line stably silencing HOTTIP, IL-6 or c-jun or that overexpressing HOTTIP or c-jun was screened out using Puromycin (2 μg/mL).

### Detection of the proliferation of T cells induced by neutrophils

Anti-CD3/anti-CD28 tetramer antibody (STEMCELL Technologies, Vancouver, BC, Canada) was used to coat the 96-well plates. IL-2 (20 IU/mL) was added to the collected CD3^+^ T cells. CD3^+^ T cells labeled by Carboxyfluorescein succinimidyl ester (CFSE; S1076, Beijing Solabio Life Sciences Co., Ltd., Beijing, China) were co-cultured with the isolated neutrophils in RPMI-1640 with 5% CO_2_ at 37 °C. Finally, flow cytometry was used to measure the content of CFSE [17].

### Determination of IL-6 secretion

The serum or OC culture medium supernatant was transferred into a fresh eppendorf (EP) tube, and then centrifuged at 8000 rpm for 8 min. The content of IL-6 was determined with the application of the IL-6 enzyme-linked immunosorbent assay (ELISA) kit (ab178013, Abcam Inc., Cambridge, MA, USA). The 96-well plate was added with 100 μL of coating solution containing IL-6 and sealed at 4 °C overnight. Afterwards, the cells in each well were incubated with 200 μL ELISA diluent for 1 h at room temperature. Next, 50 μL ELISA diluent was used to dilute 50 μL standard products, a total of 8 wells. The remaining wells were sealed with 100 μL isolated serum at room temperature for 2 h, with 3 parallel wells set up. Then, the sample was probed with the secondary antibody (100 μL) for 1 h, then reacted with ELISA diluent for 40 min, and developed with 100 μL chromogenic solution for 5–10 min devoid of light at room temperature. Finally, 50 μL of termination solution was added into each well. The optical density (OD) value was measured at 450 nm using a microplate reader [18].

### RNA isolation and quantification

The total RNA of tissues or cells was extracted using the Trizol kit (15596–018, Beijing Solabio Life Sciences Co., Ltd., Beijing, China), and the concentration of RNA was determined. The primers used in this study were synthesized by Takara Biotechnology (Dalian, Liaoning, China) (Table 2). The reverse transcription was performed with 25 μL assay system based on the instructions of the complementary DNA (cDNA) reverse transcription kit (K1622, Reanta Co., Ltd., Beijing, China). The cDNA obtained was diluted to 50 ng/μL for the following RT-qPCR, which was carried out on the fluorescence quantitative polymerase chain reaction instrument (ViiA 7, DAAN Gene Co., Ltd. of Sun Yat-sen University, Guangzhou, Guangdong, China). Glyceraldehyde-3-phosphate dehydrogenase (GAPDH) was used as an internal reference. The fold changes were calculated by means of the relative quantification (2^-ΔΔCt^ method).

**Table 2:** Primer sequences for RT-qPCR

Primer sequence	Forward (5′ - 3′)	Reverse (5′ - 3′)
HOTTIP	CCTAAAGCCACGCTTCTTTG	TGCAGGCTGGAGATCCTACT
GAPDH	ACAACAGCCTCAAGATCATCAG	GGTCCACCACTGACACGTTG

*RT-qPCR* reverse transcription quantitative polymerase chain reaction, *HOTTIP* HOXA transcript at the distal tip, *GAPDH* glyceraldehyde-3-phosphate dehydrogenase

### Fractionation of nuclear/cytoplasmic RNA

The experiment was conducted in accordance to the PARIS™ Kit Protein and RNA Isolation System (Life Technologies, Carlsbad, CA, USA). In brief, OC cells were collected, washed with PBS, trypsinized and centrifuged (500 g) 5 min at 4 °C. The cell precipitate was rinsed with PBS, after which the supernatant was discarded. Next, 500 μL of cell fractionation Buffer was added to the cells and dissociated gently, followed by standing on ice for 5–10 min. Following 5 min of centrifugation at 4 °C, 500 g, the supernatant (i.e. cytoplasm) was removed into 2 mL aseptic and enzyme-free tube, followed by additional centrifugation at 4 °C, 500 g for 5 min. The precipitate obtained (i.e. nucleus) was added with 500 μL Cell Fractionation Buffer and mixed by flicking. Next, 500 μL 2 × Lysis/binding solution was dropped to the mixture, mixed well following gentle dissociation, and subsequently allowed to stand on ice. Following the removal of the supernatant, precooled 500 μL Cell Disruption Buffer was added and whirled violently for complete mixture. Thereafter, 500 μL absolute ethanol was added and mixed by gentle dissociation. The absorption column was put into collection tubes, after which 700 μL Wash solution I was added followed by 30-s centrifugation at 12000 g and the removal of the supernatant. The liquid in the collection tubes was harvested. Similarly, 500 μL Wash solution 2/3 was added and centrifugation was carried out at 12000 g for 30 s, after which the supernatant was withdrawn and the liquid in the tubes was collected. Next, the empty column was rotated at a maximum speed for 1 min, and the collection tubes were discarded. The adsorption column was loaded into a new collection tube, and was eluted by the addition of 40 μL Elution solution (water bath to 95 °C in advance) and 30 s of centrifugation at 12000 g, followed by further elution by the addition of 10 μL Elution solution. Finally, reverse transcription quantitative polymerase chain reaction (RT-qPCR) was carried out to determine the expression of HOTTIP in cytoplasm (with 12S rRNA regarded as the loading control) and nucleus (with 45S rRNA regarded as the loading control) (Table 3).

**Table 3:** Primer sequences for fractionation of nuclear/cytoplasmic RNA

Gene	Sequence
45S rRNA	F: 5′-GTGCCCTCACGTGTTTCACTTT-3′
	R: 5′-TAGGAGACAAACCTGGAACGCT-3′
12S rRNA	F: 5′-TCGATAAACCCCGCTCTACCT-3′
	R: 5′-TGGCTACACCTTGACCTAACGTT-3′

*F* forward, *R* reverse

### Dual luciferase reporter gene assay

The National Center for Biotechnology Information (NCBI) database (http://www.ncbi.nlm.nih.gov/gene) was adopted for the retrieval of the promoter sequence and gene sequence of IL-6. The promoter region of IL-6 gene was cloned into the pmirGLO vector (Promega Corporation, Madison, WI, USA), and the wild type (wt) pmirGLO-IL-6 prom vector (IL-6-prom-wt vector). The plasmids were co-transfected with the renilla luciferase expression vector pRL-TK (TaKaRa Biotechnology Co. Ltd., Liaoning, China) in accordance with the instructions provided on the lip2000. The cells were then transfected with plasmids of IL-6-prom-wt + NC, IL-6 prom wt + shRNA (sh)-c-jun + NC, IL-6 prom wt + pLV-EGFP-N-HOTTIP, IL-6 prom wt + c-jun, IL-6 prom wt + pLV-EGFP-N-HOTTIP + c-jun and IL-6 prom wt + sh-c-jun + pLV-EGFP-N-HOTTIP. Following transfection, the cells were cultured for 24 h, and Dual-Luciferase Reporter Assay System (Promega Corporation, Madison, WI, USA) was used to determine the dual luciferase activity in each group, with renilla luciferase pRL-TK as loading control (TaKaRa, Biotechnology Co. Ltd., Liaoning, China).

### Chromatin immunoprecipitation (ChIP) assay

The binding sites between c-jun and IL-6 were predicted using the JASPAR website (http://jaspar.genereg.net/) (site 1: GCTATGATGCAATT; site 2: TGGATGACCTCAC). The cells were fixed by formaldehyde for 10 min to produce DNA-protein crosslinking. The DNA was sonicated for 10 s (15 times at an interval of 10 s). Following centrifugation at 12000×g at 4 °C for 10 min, the supernatant was collected and divided into two tubes, both of which underwent incubation overnight with rabbit antibody against c-jun (#9165, Cell Signaling Technologies, Beverly, MA, USA) or IgG (NC, ab109489, 1:300, Abcam Inc., Cambridge, MA, USA) at 4 °C. Protein Agarose/Sepharose was used to precipitate DNA-protein complex. The nonspecific complex was then washed, and the crosslinking was reversed at 65 °C overnight. The DNA fragment was purified and extracted by phenol/chloroform. The site 1 primers [forward (F) sequence: 5′-TGCACTCCCATTTGTCTGGC-3′; reverse (R) sequence: 5′-TGAATACGTGACCAGAAAAGGCT-3′] and the sequences of site 2 primers (F: 5′-ACACCAAAGAATCCCACCGC-3′; R: 5′-ATTTCAGGACCCGCCTGTTG-3′) were designed. The length of the amplified product was 130 bp and 100 bp, respectively, and the transcription start sites (TSS) were 1846 bp and 27 bp. The sequence far from the promoter region of IL-6 gene was also amplified as the NC (F: 5′-ACCACTGTACAGATCCAGCAG-3′, R: 5′-GGGTACCCAGAAAGCCTGGAC-3′) with the length of 89 bp, and the TSS of 3867 bp. RT-qPCR was carried out using the primers. The purified DNA obtained by overexpression of HOTTIP was the same as the above described with the site 2 primer used as the primers for RT-qPCR.

### RNA-binding protein immunoprecipitation (RIP)

The cells were treated with radioimmunoprecipitation assay (RIPA) lysis buffer and then the lysate was isolated. The magnetic beads were incubated with rabbit anti-human c-jun (#9165, 1:100, Cell Signaling Technologies, Beverly, MA, USA) to construct the magnetic bead-c-jun antibody complex. The complex was then mixed with the lysate. Subsequently, isolation and purification of RNA from the complex were conducted.

### Western blot analysis

After the treatment with the RIPA lysis buffer containing phenylmethanesulfonyl fluoride (PMSF) and phosphatase inhibitor in an ice-bath for 30 min, the proteins were isolated by centrifugation at 12000 rpm at 4 °C for 30 min. The protein concentration was determined with the use of the bicinchoninic acid (BCA) kit. Following separation by 10% SDS-polyacrylamide gel electrophoresis (PAGE), the proteins were transferred onto the polyvinylidene fluoride (PVDF) membrane by a wet transfer method. The membrane was then blocked with 5% skimmed milk at room temperature for 1 h and incubation was carried out with diluted rabbit primary antibodies: IL-6 antibody (ab9324), PD-L1 antibody (ab228415), STAT3 antibody (ab68153), p-STAT3 antibody (ab76315), and mouse anti-β-actin antibody (ab8226, 1:1000). All of the aforementioned antibodies were purchased from Abcam Inc. (Cambridge, MA, USA). After being washed, the membrane was incubated with secondary antibody horseradish peroxidase (HRP)-conjugated goat anti-mouse IgG or goat anti-rabbit IgG (Transgene Biotech, Beijing, China) for 1 h. The immunocomplexes on the membrane were visualized using enhanced chemiluminescence (ECL) fluorescent detection kit (BB-3501, Amersham Pharmacia, Piscataway, NJ, USA) and band intensities were quantified using Bio-Rad image analysis system (Bio-Rad Laboratories, Hercules, CA, USA) and Quantity One v4.6.2 software. The ratio of the gray value of the target protein band to that of β-actin band was regarded as the relative protein expression.

### In vivo xenograft experiments

Twenty-five non-obese diabetic/severe combined immunodeficient (NOD/SCID) mice (5–7 weeks old; weight 18–22 g; purchased from Shanghai Lingchang, Shanghai, China) were divided into 5 groups with 5 mice in each group. All animals were allowed to acclimatize for 1 week in the specific-pathogen-free (SPF) animal facility in Tongji Hospital of Tongji University and The Sixth People’s Hospital of Yancheng City before any intervention was initiated. The mice were provided with sterile feeding and drinking water with alternated day and night for 12 h. The SKOV3 single cell suspension was prepared with the concentration of 2 × 10^6^ cells/mL and 0.2 mL of cell suspension was subcutaneously injected into the left axillary of the mice using 1 mL syringes. The mice (5 in each group) were intraperitoneally injected respectively with 100 μL PBS, neutrophils + T cells (5 × 10^6^ cells for either;^,^ 1: 1, a total of 100 μL), SKOV3-neutrophils + T cells (5 × 10^6^ cells for either; 1: 1, a total of 100 μL), SKOV3-neutrophils overexpressing HOTTIP + T cells (5 × 10^6^ cells for either; 1: 1, a total of 100 μL), or PD-L1 antibody (20 μg/mL)-treated SKOV3-neutrophils overexpressing HOTTIP and T cells (5 × 10^6^ cells for either; 1: 1, a total of 100 μL). Following inoculation, all the nude mice were kept in the SPF animal room.

The short diameter (a) and long diameter (b) of tumors were measured every 3 days using the calipers. The tumor volume was estimated using the following formula: π (a^2^ × b) / 6. Once the mice were euthanized, the tumors were weighed. A portion of the tissue was further excised for immunohistochemical staining, and another portion was used to prepare tumor-infiltrating lymphocyte (TIL) using the tumor dissociation kit (Miltenyi biotec, Bergisch Gladbach, Germany) and TIL gentleMACS dissociator (Miltenyi biotec, Bergisch Gladbach, Germany). TILs were isolated from tumor suspensions through density gradient centrifugation with gradient Percoll II solution (GE Healthcare, Pittsburgh, PA, USA). Flow cytometry was used for the analysis of IFN-γ expression in TILs [17].

### Statistical analysis

All analyses were conducted using SPSS 21.0 statistical software (IBM Corp, Armonk, NY, USA). Measurement data were expressed as mean ± standard deviation. Data with normal distribution and homogeneity of variance between two groups were compared using paired *t*-test (paired data) or unpaired *t*-test (unpaired data). One-way analysis of variance was performed for comparisons among multiple groups, followed by Tukey’s post hoc test. The repeated measures analysis of variance with Bonferroni post hoc test was applied for the comparison of data at different time points. Correlation between parameters was evaluated by Pearson’s correlation coefficient. OS was assessed using the Kaplan-Meier method and the log-rank test. *p* < 0.05 was considered a statistically significant value.

## Results

### IL-6 is highly expressed in OC tissues and positively associated with PD-L1 in neutrophils

**Fig. 1:**
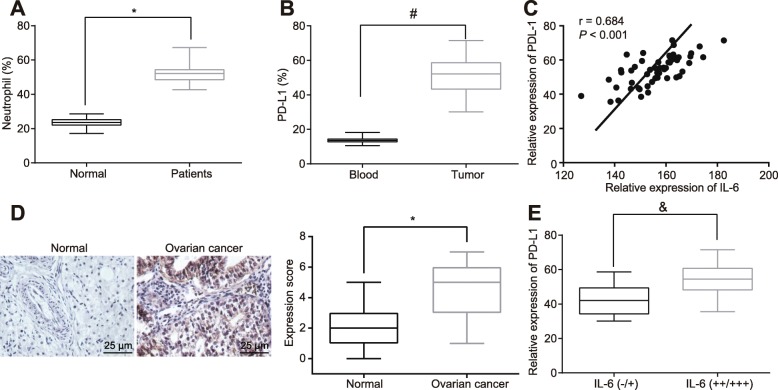
IL-6 is highly expressed in OC and positively associate PD-L1 in neutrophils. **a** The number of neutrophils in peripheral blood of OC patients and normal controls as determined by flow cytometry. **b** The expression of PD-L1 in infiltrated neutrophils and neutrophils from peripheral blood of OC patients as measured by flow cytometry. **c** The correlation between IL-6 expression in peripheral blood as detected by RT-qPCR and PD-L1 expression in infiltrated neutrophils as determined by flow cytometry analyzed by Pearson’s correlation coefficient. **d** The expression of IL-6 in OC tissues as detected by immunohistochemical staining (400 ×). **e** The Pearson’s correlation analysis between IL-6 expression determined by immunohistochemical staining and PD-L1 expression in infiltrated neutrophils determined by flow cytometry. ^*^ *p* < 0.05 vs. normal controls; ^#^ *p* < 0.05 vs. the neutrophils in peripheral blood; ^&^ *p* < 0.05 vs. the IL-6 (−/+). The measurement data were summarized as mean ± standard deviation. The unpaired *t* test was used to compare the data between two groups. The experiment was repeated 3 times independently

To evaluate the potential role of neutrophils in the immune system of OC patients, the number of neutrophils in peripheral blood collected from clinical samples was assessed by flow cytometry. Notably, OC patients presented with a greater number of neutrophils relative to normal controls (Fig. 1a, *p* < 0.05). Next, infiltrated neutrophils in OC were isolated from peripheral blood using flow cytometry and then the expression of PD-L1 in neutrophils from peripheral blood and infiltrated neutrophils was evaluated. The results revealed that PD-L1 expression in infiltrated neutrophils was elevated in comparison to that of the neutrophils from peripheral blood (Fig. 1b, *p* < 0.05), suggesting that tumor cells might be engaged in the PD-L1 expression in infiltrated neutrophils in OC.

Furthermore, in order to explore the association between the PD-L1 expression in infiltrated neutrophils and the IL-6 expression in peripheral blood of OC patients, flow cytometry and RT-qPCR were conducted to detect the expression of PD-L1 in infiltrated neutrophils and IL-6 in OC patients and normal controls. The results illustrated a significant enhancement in IL-6 expression in peripheral blood of OC patients relative to normal controls, and the higher expression IL-6 in peripheral blood of OC patients was closely associated with the higher expression of PD-L1 in infiltrated neutrophils in OC (Fig. 1c, *p* < 0.05). The results inferred that the abnormal expression of IL-6 in OC patients resulted in higher expression of PD-L1 in infiltrated neutrophils in OC. In consistent, the immunohistochemical staining exhibited that neutrophils were accumulated in the OC tissues compared to the normal fallopian tube fimbriae tissues (Fig. 1d, *p* < 0.05). Following extraction of the infiltrated neutrophils in OC, we conducted flow cytometry to measure the expression of PD-L1. Combined with the immunohistochemical staining results of the upregulated PD-L1 expression in OC tissues, a positive relationship was noted between IL-6 expression in OC tissues and PD-L1 expression in infiltrated neutrophils (Fig. 1e, *p* < 0.05). Collectively, these data suggested upregulated IL-6 in OC tissues, which might secrete to the surrounding infiltrated neutrophils, thereby contributing to PD-L1 overexpression.

### OC cells secret IL-6 to activate the STAT3/PD-L1 pathway in neutrophils and repress T cell proliferation

**Fig. 2:**
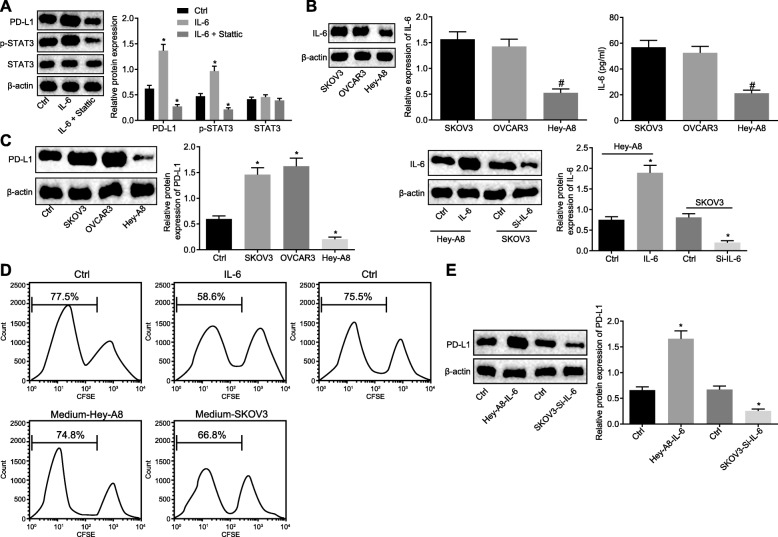
IL-6 secreted by OC cells inhibits T cell proliferation by activating the STAT3/PD-L1 pathway in neutrophils. **a** Western blot analysis of PD-L1 and STAT3 proteins as well as the extent of STAT3 phosphorylation in neutrophils added with IL-6 (3 μg/mL) and STAT3 phosphorylation inhibitor Stattic (10 μM). **b** IL-6 expression in OC cell lines: SKOV3, Hey-A8 and OVCAR3 as determined by Western blot analysis and ELISA. **c** Western blot analysis of PD-L1 protein in neutrophils added with three kinds of OC cell culture medium (on the left) and added with Hey-A8 cells treated with overexpressed IL-6 and SKOV3 cells silencing IL-6 (on the right). **d** The proliferation of CFSE-labeled T cells co-cultured with neutrophils treated with Hey-A8/IL-6 and SKOV3/si-IL-6, as examined by flow cytometry. **e** Western blot analysis of PD-L1 protein in neutrophils added with Hey-A8 and SKOV3 culture solution. ^*^ *p* < 0.05 vs. controls; ^#^ *p* < 0.05 vs. SKOV3 cell culture solution; The measurement data were summarized as mean ± standard deviation. The unpaired *t* test was used to compare the data between two groups. The one-way analysis of variance was adopted to compare the data among multiple groups, followed by Tukey’s post hoc test. The experiment was repeated 3 times independently

To discover the probable functions of IL-6 on neutrophils, IL-6 (3 μg/mL) was supplemented to neutrophils, and the expression of PD-L1 and STAT3 in addition to the extent of STAT3 phosphorylation in neutrophils were measured as well. As shown in Fig. 2a, content of PD-L1 and the extent of STAT3 phosphorylation increased (*p* < 0.05), suggesting that IL-6 could promote the extent of STAT3 phosphorylation and increase expression of PD-L1 in neutrophils. In order to verify whether IL-6 promoted expression of PD-L1 through STAT3 phosphorylation, the STAT3 phosphorylation inhibitor Stattic was applied. The results found that the content PD-L1 was not up-regulated following the addition of IL-6 (*p* < 0.05). The results showed that IL-6 stimulated the up-regulation of PD-L1 mainly by promoting phosphorylation of STAT3.

The expression of IL-6 in OC cell lines was determined. It was validated that the expression of IL-6 in SKOV3 and OVCAR3 cells were higher than that in Hey-A8 cells (Fig. 2b, *p* < 0.05). Next, cell culture solution of SKOV3, Hey-A8 and OVCAR3 was added to neutrophils and the expression of PD-L1 was evaluated using RT-qPCR and Western blot analysis. As shown in Fig. 2c, PD-L1 expression was enhanced in neutrophils cultured with SKOV3 or OVCAR3 cells versus that with Hey-A8 cells (*p* < 0.05), suggesting that OC cells with higher expression of IL-6 induced PD-L1 expression in neutrophils. For further verification, IL-6 gene was knockout in SKOV3 cells, and IL-6 was overexpressed in Hey-A8 cells. The PD-L1 expression in neutrophils was found to be decreased following culture with IL-6-knockout SKOV3 cells, while it was promoted following culture with Hey-A8 cells overexpressing IL-6 (*p* < 0.05).

The roles of IL-6-induced PD-L1 in neutrophils in T cell proliferation were subsequently explored. T cells were labeled with CFSE and co-cultured with neutrophils pre-treated with IL-6. The changes of T cell proliferation were then assessed by flow cytometry, which demonstrated that the proliferation of T cells co-cultured with neutrophils pretreated IL-6 was diminished (Fig. 2d, *p* < 0.05). To verify whether OC cells with high expression of IL-6 can inhibit proliferation of T cells by promoting expression of PD-L1 in neutrophils, the SKOV3 cell culture medium was added to neutrophils, and co-cultured with T cells. The proliferation ability of T cells was assessed by flow cytometry, which revealed reduced proliferation ability of T cells (*p* < 0.05). In addition, after the SKOV3 cell culture medium was replaced by the Hey-A8 medium, no difference was observed in T cell proliferation (Fig. 2d, *p* > 0.05). Western blot assay (Fig. 2e) showed that the expression of PD-L1 was significantly increased in neutrophils after Hey-A8 cell culture were added to neutrophils. After SKOV3 cell culture was added to neutrophils, the expression of PD-L1 in neutrophils was significantly reduced. Therefore, we may conclude that the OC cells up-regulated PD-L1 expression in neutrophils by secreting IL-6, thus inhibiting T cell proliferation.

### HOTTIP promotes IL-6 transcription by regulating transcription factor c-jun

**Fig. 3:**
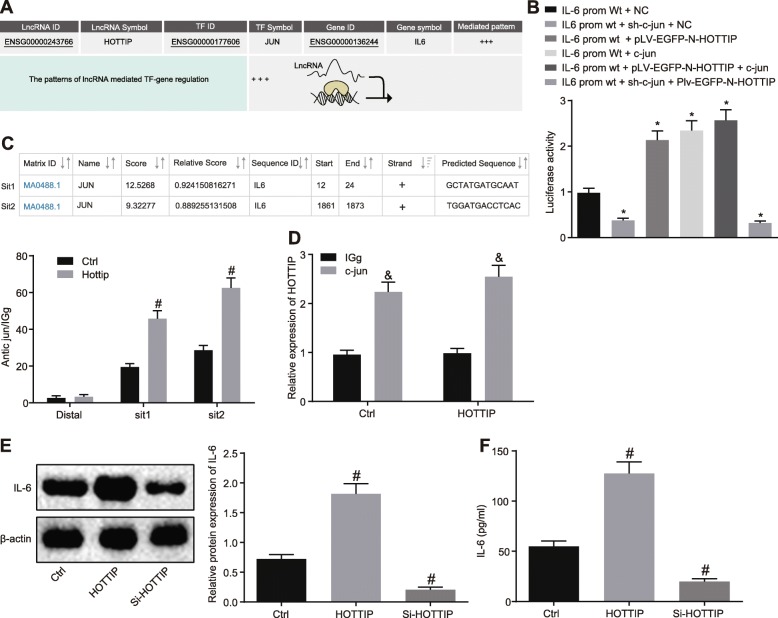
HOTTIP stimulates IL-6 transcription via regulating c-jun. **a** The relationship between HOTTIP, IL-6 and c-jun predicted by LNCmap. **b** The binding relationship between HOTTIP and IL-6, as well as between c-jun and IL-6 confirmed by dual luciferase reporter gene assay. **c** The binding sites between c-jun and IL-6 promoter predicted and verified by ChIP assay. **d** The interaction between c-jun and HOTTIP validated by RIP assay. **e** Western blot analysis of IL-6 protein in SKOV3 cells overexpressing or silencing HOTTIP. **f** The secretion of IL-6 in SKOV3 cells overexpressing or silencing HOTTIP as measured by ELISA. ^*^ *p* < 0.05 vs. cells treated with IL-6-prom-wt plus NC; ^#^ *p* < 0.05 vs. cells without treatment; ^&^ *p* < 0.05 vs. cells treated with IgG; The measurement data were summarized as mean ± standard deviation. The unpaired *t* test was used to compare the data in two groups. The one-way analysis of variance was adopted to compare the data among multiple groups, followed by Tukey’s post hoc test. The experiment was repeated 3 times independently

Next, the mechanism of IL-6 overexpression in OC was studied. The bioinformatics prediction website indicated that HOTTIP may promote IL-6 transcription by binding to c-jun (Fig. 3a). Fractionation of nuclear/cytoplasmic RNA displayed that HOTTIP was predominantly localized in nucleus (Additional file 1: Figure S1). Dual luciferase reporter gene assay revealed that both c-jun and HOTTIP could increase the activity of IL-6 promoter (Fig. 3b, *p* < 0.05). Moreover, two binding sites between c-jun and IL-6 promoter with the highest score were predicted, which were further conformed by the ChIP assay. In addition, the effect of HOTTIP on the binding of c-jun and IL-6 promoter was evaluated. The results noted that HOTTIP enhanced the binding of c-jun and IL-6 promoter (Fig. 3c, *p* < 0.05). Moreover, the interaction between c-jun and HOTTIP was verified in SKOV3 cells by RIP assay (Fig. 3d, *p* < 0.05). When HOTTIP was overexpressed in SKOV3 cells, the expression and secretion levels of IL-6 were enhanced; however, the expression and secretion levels of IL-6 were reduced following HOTTIP silencing (Fig. 3e & f, *p* < 0.05), suggesting HOTTIP can promote expression of IL-6. Altogether, HOTTIP could augment transcription of IL-6 via transcription factor c-jun.

### Upregulation of HOTTIP restrains T cell proliferation by promoting IL-6

**Fig. 4:**
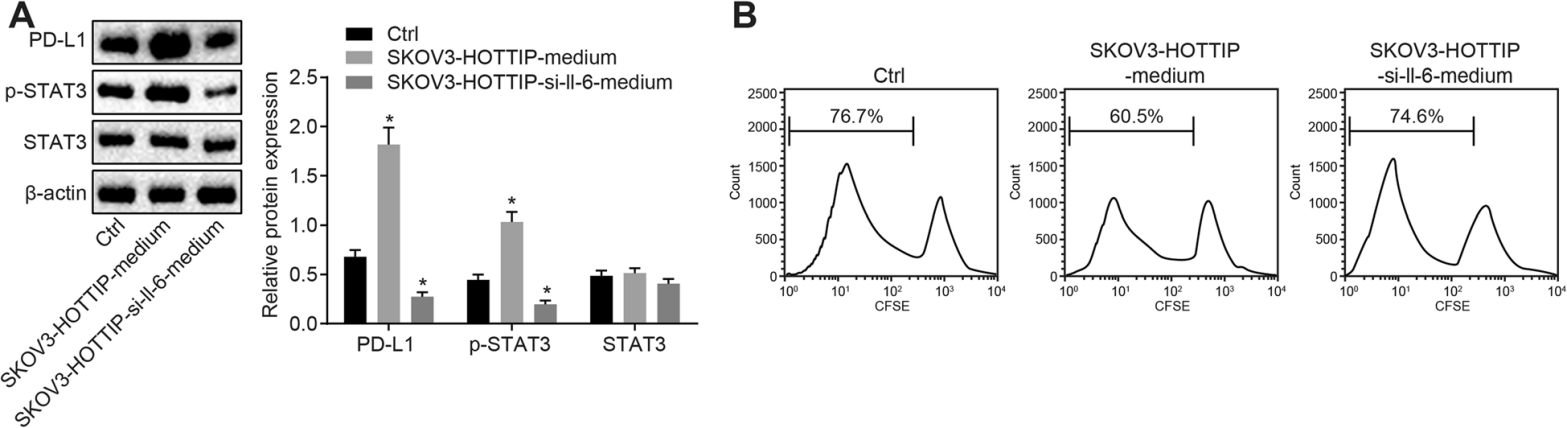
HOTTIP promotes neutrophils induced-suppression on T cell proliferation by enhancing IL-6 expression. **a** Western blot analysis of STAT3 and PD-L1 proteins as well as the extent of STAT3 phosphorylation in SKOV3 cells overexpressing HOTTIP with or without IL-6 silencing. **b** The ability of proliferation of CFSE-labeled T cells co-cultured with neutrophils added with SKOV3 cells overexpressing HOTTIP with or without IL-6 silencing, as determined by flow cytometry. ^*^ *p* < 0.05 vs. the cells without treatment. The measurement data were summarized as mean ± standard deviation. The one-way analysis of variance was adopted for comparison among multiple groups, followed by Tukey’s post hoc test. The experiment was repeated 3 times independently

To investigate the effects of HOTTIP on the expression of PD-L1 in neutrophils and the proliferation of T cells in OC, HOTTIP expression was enforced in SKOV3 cells and then the cell culture medium was supplemented to neutrophils. Next, Western blot analysis was conducted to detect the protein expression of STAT3 and PD-L1 as well as the extent of STAT3 phosphorylation in neutrophils, which suggested that the protein expression of PD-L1 and the extent of STAT3 phosphorylation were elevated in neutrophils treated with SKOV3 cell culture medium (Fig. 4a, *p* < 0.05). Next, the neutrophils added with SKOV3 cells both overexpressing HOTTIP and silencing IL-6 were co-cultured with T cells for a time, after which the T cell proliferation was assessed using flow cytometry. It was implied that the proliferation of T cells was repressed by neutrophils cultured with SKOV3 cell culture medium (Fig. 4b, *p* < 0.05).

Afterwards, the mechanism of suppression of T cell proliferation by HOTTIP was explored. In OC cells treated with overexpressed HOTTIP, IL-6 was silenced. After a period of culture, the culture medium was obtained and added to the neutrophils. The protein expression of PD-L1 and STAT3 as well as the extent of STAT3 phosphorylation in neutrophils was subsequently estimated using Western blot analysis. No significant changes were detected in PD-L1 and STAT3 protein expression and the extent of STAT3 phosphorylation in neutrophils added with SKOV3 cells both overexpressing HOTTIP and silencing IL-6 (Fig. 4a, *p* > 0.05), indicating that HOTTIP augmented PD-L1 expression in neutrophils mainly through IL-6. Meanwhile, the neutrophils added with SKOV3 cells both overexpressing HOTTIP and silencing IL-6 were dropped into the T cells labeled by CFSE and then co-cultured. Flow cytometry was followed to assess the proliferation ability of T cells. The results demonstrated no remarkable difference in the proliferation ability of T cells (Fig. 4b, *p* < 0.05). The aforementioned results suggest that HOTTIP exerted a promoting role in neutrophils induced inhibition of T cell proliferation by promoting IL-6 in OC cells.

### Upregulation of HOTTIP increases PD-L1 expression and inhibits T cell-mediated tumor immunotherapy

**Fig. 5:**
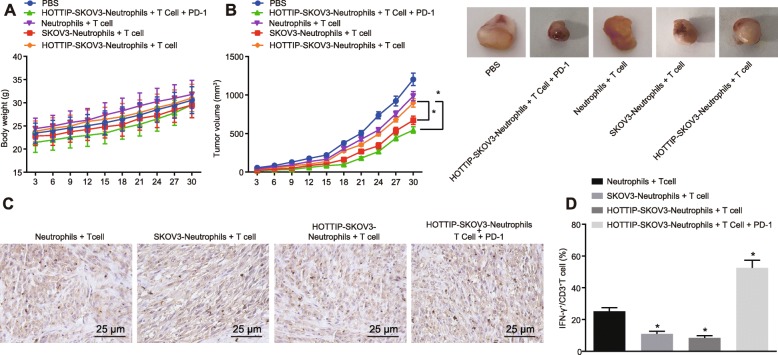
HOTTIP enhances PD-L1 expression in neutrophils, thereby contributing to immune escape of OC cells. The mice (5 in each group) were intraperitoneally injected respectively with 100 μL PBS, neutrophils + T cells (5 × 10^6^ cells for either;^,^ 1: 1, a total of 100 μL), SKOV3-neutrophils + T cells (5 × 10^6^ cells for either; 1: 1, a total of 100 μL), SKOV3-neutrophils overexpressing HOTTIP + T cells (5 × 10^6^ cells for either; 1: 1, a total of 100 μL), or PD-L1 antibody (20 μg/mL)-treated SKOV3-neutrophils overexpressing HOTTIP and T cells (5 × 10^6^ cells for either; 1: 1, a total of 100 μL). **a** The body weight of mice after treatment. **b** The tumor volume of mice after different treatment. **c** Immunohistochemical staining of CD3^+^ T cell infiltration (400 ×). **d** The expression of IFN-γ^+^ in CD3 T^+^ cells as detected by flow cytometry. ^*^ *p* < 0.05 vs. the SKOV3-neutrophils + T cells. The measurement data were summarized as mean ± standard deviation. The one-way analysis of variance was adopted to compare the data among multiple groups, followed by Tukey’s post hoc test. Repeated measures analysis of variance was applied to compare the data at different time points, followed by Bonferroni post hoc test. *n* = 5. The experiment was repeated 3 times independently

The effect of HOTTIP on T cell immunity was evaluated in vivo. At the 10th d of tumorigenesis, the neutrophils treated with the medium from the normal SKOV3 cells or SKOV3 cells overexpressing HOTTIP were intraperitoneally injected with PBS and intraperitoneally injected with CD3^+^ T cells into the established NOD/SCID mice with or without PD-L1 antibody. Subsequently, the body weight of the mice and the tumor volume were recorded. As revealed in Fig. 5a, no significant difference was visible in body weight upon different treatments (*p* > 0.05). However, the tumor volume of mice injected with neutrophils treated with SKOV3 cells overexpressing HOTTIP was much heavier than that in neutrophils treated with normal SKOV3 cells (Fig. 5b, *p* < 0.05). Then all the tumors were excised and the percentage of CD3^+^ T cells was measured by immunohistochemical staining. A decline of the infiltrated CD3^+^ T cells was observed in mice injected with neutrophils added with SKOV3 cells overexpressing HOTTIP while CD3^+^ T cells increased following the addition of PD-L1 antibody (Fig. 5c, *p* < 0.05). Meanwhile, the IFN-γ expression in CD3^+^ T cells was detected through flow cytometry to determine the immune activity of T cells. The results showed that the expression of IFN-γ^+^ in CD3^+^ T cells was relatively lower in neutrophils added with SKOV3 cells overexpressing HOTTIP, while the results was restored by adding PD-L1 antibody (Fig. 5d, *p* < 0.05). These results indicated that HOTTIP could promote the expression of PD-L1 in neutrophils, while suppressing T cell immunity in vivo.

### HOTTIP is highly expressed in OC tissues and positively correlates with PD-L1 in neutrophils

**Fig. 6:**
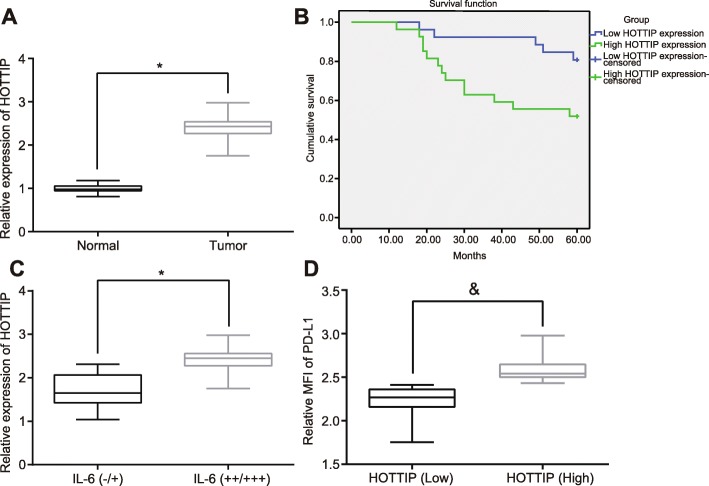
Overexpression of HOTTIP in OC tissues enhances the expression of PD-L1 in neutrophils. **a** HOTTIP expression in OC tissues and normal fallopian tube fimbriae tissues as determined by RT-qPCR. **b** Analysis of the relationship between expression of HOTTIP and the OS of patients with OC by Kaplan-Meier analysis. **c** The correlation between the expression of HOTTIP and IL-6. **d** The correlation between the expression of HOTTIP and PD-L1. ^*^ *p* < 0.05 vs. normal fallopian tube fimbriae tissues; ^#^ *p* < 0.05 vs. the IL-6(−/+); ^&^ *p* < 0.05 vs. patients with lower expression of HOTTIP. The measurement data were summarized as mean ± standard deviation. The unpaired *t* test was used to compare the data between two groups. The experiment was repeated 3 times independently

To confirm the HOTTIP expression in OC tissues as well as the association with prognosis of OC patients, initially, the expression of HOTTIP was evaluated in OC tissues and normal fallopian tube fimbriae tissues using RT-qPCR. The results found that HOTTIP expression was elevated in OC tissue relative to that in normal fallopian tube fimbriae tissues (Fig. 6a, *p* < 0.05). Next, we conducted clinical follow-up to analyze the association of HOTTIP expression with prognosis of OC. It was revealed that the OS of patients with higher expression of HOTTIP (cut off value was 3.22) was far shorter than that of OC patients with lower HOTTIP expression (Fig. 6b, *p* < 0.05).

Subsequent correlation analysis revealed a positive correlation between expression of IL-6 and expression of HOTTIP (Fig. 6c, *p* < 0.05). Moreover, HOTTIP could also positively associate with the PD-L1 expression in neutrophils (Fig. 6d, *p* < 0.05). The aforementioned data supported a strong and positive correlation between HOTTIP expression and PD-L1 expression in neutrophils.

## Discussion

Recent studies have reported that HOTTIP plays critical roles in the progression of several types of human malignancies, such as nasopharyngeal cancer [19], oral tongue squamous cell carcinoma [20] as well as breast cancer [21]. In this expanding field, much remains unknown regarding the function of HOTTIP in the development of OC and the underlying mechanism. Hence, the present study aims at demonstrating the possible mechanism by which HOTTIP was implicated in OC development. Collectively, HOTTIP could elicit the secretion of IL-6 and up-regulate PD-L1 expression in neutrophils, so as to suppress the activity of T cells, ultimately accelerating immune escape of OC cells.

Our findings indicated a robust expression of HOTTIP and IL-6 in OC tissues. Similarly, HOTTIP was up-regulated in gastric cancer (GC) cell lines [22]. Also, HOTTIP expression was notably higher in OC tissue samples in comparison to adjacent normal tissue samples [23]. In addition, OC patients presented with consistently elevated expression of IL-6 [24]. Furthermore, IL-6 has been noted to be in a positive correlation with PD-L1, which was expressed intensely in OC cells [25], which was consistent with our findings.

A pull-down assay confirmed putative binding sites of lncRNA DILC within the IL-6 promoter in HCC [26]. Knockdown of MALAT1 suppresses the phosphorylation of c-jun N-terminal kinase 1 (JNK1) in OC cells [27]. Consistently, our results revealed that HOTTIP could induce the release of IL-6 via c-jun. In addition, IL-6 abrogates T cell proliferation by promoting the extent of STAT3 phosphorylation through the enhancement of PD-L1 expression in neutrophils. Downregulation of IL-6/JAK/STAT3 pathway activation subsequently reduces the expression of PD-L1 in non-small cell lung cancer [15]. Cancer-associated fibroblasts induce PD-L1^+^ neutrophils by promoting the IL-6-STAT3 pathway that fosters immune suppression in HCC [28]. IL-6 mediates STAT3 activation in tumor and tumor progression, and IL-6 neutralization reduces STAT3 activity in vivo [29]. The aforementioned findings were in line with our findings that the addition of STAT3 inhibitor Stattic eliminated the promoting effects of IL-6 on PD-L1 expression. Notably, the IL-6/STAT3 axis can simultaneously result in the elevation of the growth of immunosuppressive cells or modify the balance of T-cell subsets [30]. The decline in IL-6 may be suggestive of the contrasting role of effector T cells and oppressive myeloid cells [31], and our study demonstrated that neutrophils treated with OC cells with the up-regulation of HOTTIP plus IL-6 silencing did not result in a significant change in T-cell proliferation.

Moreover, enhanced HOTTIP has been observed to elevate PD-L1 expression and result in increased immunosuppression, as demonstrated by suppressed T cell proliferation, IFN-γ expression as well as elevated tumor weigh in nude mice. Previous data revealed that the expression of HOTTIP and PD-L1 was simultaneously dysregulated in non-small cell lung cancer [32]. LncRNA MALAT1 promotes tumorigenesis and immune escape of diffuse large B cell lymphoma by promoting PD-L1 capable of enhancing the tumorigenesis and immune escape abilities of cancers [33]. In GC, PD-L1 restoration in infiltrated neutrophils played an inhibitory role on IFN-γ expression and T cell proliferation ability, further highlighting the significance of PD-L1 in tumor-related immunosuppression [17]. Furthermore, PD-L1 is elevated on the CD44^+^ subpopulation, and preferentially expressed PD-L1 can, at least partially, induce the suppression of T cell proliferation through these cells [34]. Although PD-L1 is not expressed in the majority of the OC cell lines, PD-L1 expression in response to IFN-γ is detected in OC cells in patient ascites, and PD-L1 knockdown in tumor cells can diminish the tumor-promoting effect of IFN-γ [16]. In recent years, the immunotherapy based on PD-1 impairment has profoundly improved the survival of melanoma patients in advanced stage that had restricted therapeutic options [35]. Some partial response patients with GC exhibited diminished effector regulatory T cells and PD-1 expression through CD8^+^ T cells in TILs, which may reflect reduced tumor burdens [36].

## Conclusion

**Fig. 7:**
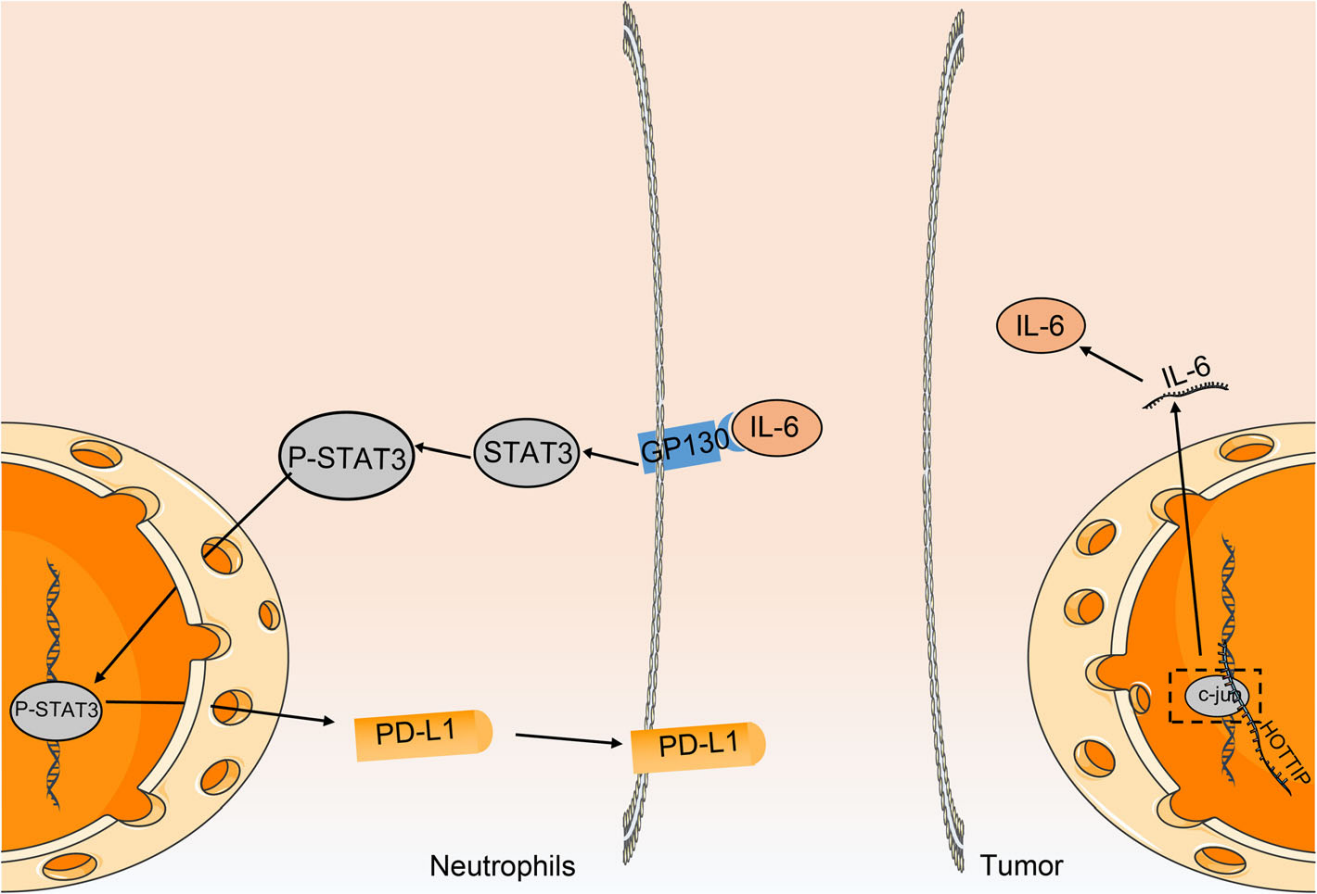
The schematic diagram depicts the regulatory mechanism of HOTTIP in the immune escape of OC cells. In OC cells, high expression of HOTTIP can enhance the expression and secretion of IL-6 from OC cells by promoting the transcription of IL-6. The secreted IL-6 stimulates the expression of PD-L1 by binding to the IL-6 receptor on the surface of neutrophils around the cancer cells and then activates the STAT3 pathway, thus increasing the expression of PD-L1 on the surface of neutrophils and inhibiting the activity of T cells, which further promotes apoptosis, thus facilitating the immune escape of OC cells

Altogether, our study identified that HOTTIP can potentially accelerate immune escape of OC cells via enhancement of IL-6-dependent PD-L1 expression (Fig. 7). This presents a novel mechanism for the role of HOTTIP in regulating immune escape of OC cells and provides a potential target for OC therapy. However, if experimental funds are permitted, further studies will be required based on the syngeneic model that is more ideal to demonstrate anti-tumor immune response to explain the specific underlying mechanism by which HOTTIP/IL-6/PD-L1 network influences OS.

## Additional file

Additional file 1: **Figure S1.** The localization and transfection efficiency of HOTTIP. A, Subcellular localization of HOTTIP in cells as measured using Fractionation of nuclear/cytoplasmic RNA. B and C, Silencing efficiency of HOTTIP in the nucleus as detected by RT-qPCR. (EPS 505 kb)

## Data Availability

The datasets generated/analysed during the current study are available.

## Abbreviations

ATCC: American Type Culture Collection
cDNA: Complementary DNA
ChIP: Chromatin immunoprecipitation
DAB: Diaminobenzidine
DMEM: Dulbecco’s modified Eagle’s medium
ECL: Enhanced chemiluminescence
ELISA: Enzyme-linked immunosorbent assay
EP: Eppendorf
F: Forward
FBS: Fetal bovine serum
GAPDH: Glyceraldehyde-3-phosphate dehydrogenase
HCC: Hepatocellular carcinoma
HOTTIP: HOXA transcript at the distal tip
HRP: Horseradish peroxidase
IFN: Interferon
IgG: Immunoglobulin G
IL-6: Interleukin-6
LncRNA: Long non-coding RNA
NC: Negative control
NCBI: National Center for Biotechnology Information
NOD/SCID: Nonobese diabetic/severe combined immunodeficient
OC: Ovarian cancer
OD: Optical density
OS: The overall survival
PAGE: Polyacrylamide gel electrophoresis
PBMCs: Peripheral blood mononuclear cells
PBS: Phosphate-buffered saline
PD-L1: Program death ligand 1
PMSF: Phenylmethanesulfonyl fluoride
PVDF: Polyvinylidene fluoride
R: Reverse
RIP: RNA-binding protein immunoprecipitation
RIPA: Radioimmunoprecipitation assay
RPMI: Roswell Park Memorial Institute
SDS: Sodium dodecyl sulfate
sh: Short hairpin RNA
si: Small interfering RNA
SPF: Specific-pathogen-free
TIL: Tumor-infiltrating lymphocyte
Treg: Regulatory T
TSS: Transcription start sites
wt: Wild type

## References

[1] Kurman RJ, Shih IM (2010). The origin and pathogenesis of epithelial ovarian cancer: a proposed unifying theory. Am J Surg Pathol.

[2] Masucci GV, Cesano A, Eggermont A, Fox BA, Wang E, Marincola FM, Ciliberto G, Dobbin K, Puzanov I, Taube J (2017). The need for a network to establish and validate predictive biomarkers in cancer immunotherapy. J Transl Med.

[3] Marth C, Wieser V, Tsibulak I, Zeimet AG (2019). Immunotherapy in ovarian cancer: fake news or the real deal?. Int J Gynecol Cancer.

[4] Looi CK, Chung FF, Leong CO, Wong SF, Rosli R, Mai CW (2019). Therapeutic challenges and current immunomodulatory strategies in targeting the immunosuppressive pancreatic tumor microenvironment. J Exp Clin Cancer Res.

[5] Hamanishi J, Mandai M, Abiko K, Matsumura N, Baba T, Yoshioka Y, Kosaka K, Konishi I (2011). The comprehensive assessment of local immune status of ovarian cancer by the clustering of multiple immune factors. Clin Immunol.

[6] Klink M, Jastrzembska K, Nowak M, Bednarska K, Szpakowski M, Szyllo K, Sulowska Z (2008). Ovarian cancer cells modulate human blood neutrophils response to activation in vitro. Scand J Immunol.

[7] He G, Zhang H, Zhou J, Wang B, Chen Y, Kong Y, Xie X, Wang X, Fei R, Wei L (2015). Peritumoural neutrophils negatively regulate adaptive immunity via the pd-l1/pd-1 signalling pathway in hepatocellular carcinoma. J Exp Clin Cancer Res.

[8] Dietl J, Engel JB, Wischhusen J (2007). The role of regulatory t cells in ovarian cancer. Int J Gynecol Cancer.

[9] Zhou SL, Zhou ZJ, Hu ZQ, Huang XW, Wang Z, Chen EB, Fan J, Cao Y, Dai Z, Zhou J (2016). Tumor-associated neutrophils recruit macrophages and t-regulatory cells to promote progression of hepatocellular carcinoma and resistance to sorafenib. Gastroenterology..

[10] Spizzo R, Almeida MI, Colombatti A, Calin GA (2012). Long non-coding rnas and cancer: a new frontier of translational research?. Oncogene..

[11] Ren C, Li X, Wang T, Wang G, Zhao C, Liang T, Zhu Y, Li M, Yang C, Zhao Y (2015). Functions and mechanisms of long noncoding rnas in ovarian cancer. Int J Gynecol Cancer.

[12] Yim GW, Kim HJ, Kim LK, Kim SW, Kim S, Nam EJ, Kim YT (2017). Long non-coding rna hoxa11 antisense promotes cell proliferation and invasion and predicts patient prognosis in serous ovarian cancer. Cancer Res Treat.

[13] Sun Y, Zeng C, Gan S, Li H, Cheng Y, Chen D, Li R, Zhu W (2018). Lncrna hottip-mediated hoxa11 expression promotes cell growth, migration and inhibits cell apoptosis in breast cancer. Int J Mol Sci.

[14] Fielding CA, McLoughlin RM, McLeod L, Colmont CS, Najdovska M, Grail D, Ernst M, Jones SA, Topley N, Jenkins BJ (2008). Il-6 regulates neutrophil trafficking during acute inflammation via stat3. J Immunol.

[15] Zhang N, Zeng Y, Du W, Zhu J, Shen D, Liu Z, Huang JA (2016). The egfr pathway is involved in the regulation of pd-l1 expression via the il-6/jak/stat3 signaling pathway in egfr-mutated non-small cell lung cancer. Int J Oncol.

[16] Abiko K, Matsumura N, Hamanishi J, Horikawa N, Murakami R, Yamaguchi K, Yoshioka Y, Baba T, Konishi I, Mandai M (2015). Ifn-gamma from lymphocytes induces pd-l1 expression and promotes progression of ovarian cancer. Br J Cancer.

[17] Wang TT, Zhao YL, Peng LS, Chen N, Chen W, Lv YP, Mao FY, Zhang JY, Cheng P, Teng YS (2017). Tumour-activated neutrophils in gastric cancer foster immune suppression and disease progression through gm-csf-pd-l1 pathway. Gut..

[18] Nilsson MB, Langley RR, Fidler IJ (2005). Interleukin-6, secreted by human ovarian carcinoma cells, is a potent proangiogenic cytokine. Cancer Res.

[19] Shen M, Li M, Liu J (2019). Long noncoding rna hottip promotes nasopharyngeal cancer cell proliferation, migration, and invasion by inhibiting mir-4301. Med Sci Monit.

[20] Mu M, Li Y, Zhan Y, Li X, Zhang B (2018). Knockdown of hoxa transcript at the distal tip suppresses the growth and invasion and induces apoptosis of oral tongue squamous carcinoma cells. Onco Targets Ther.

[21] Han S, Jin X, Liu Z, Xing F, Han Y, Yu X, He G, Qiu F. The long non-coding RNA HOTTIP promotes breast cancer cell migration, invasion, and epithelial-mesenchymal transition via Wnt/β-catenin pathway. Biochem Cell Biol. 2019. 10.1139/bcb-2018-0313. 10.1139/bcb-2018-0313 30676763

[22] Chang S, Liu J, Guo S, He S, Qiu G, Lu J, Wang J, Fan L, Zhao W, Che X (2016). Hottip and hoxa13 are oncogenes associated with gastric cancer progression. Oncol Rep.

[23] Zou Ting, Wang Ping Ling, Gao Yan, Liang Wen Tong (2019). Long noncoding RNA HOTTIP is a significant indicator of ovarian cancer prognosis and enhances cell proliferation and invasion. Cancer Biomarkers.

[24] Penson RT, Kronish K, Duan Z, Feller AJ, Stark P, Cook SE, Duska LR, Fuller AF, Goodman AK, Nikrui N (2000). Cytokines il-1beta, il-2, il-6, il-8, mcp-1, gm-csf and tnfalpha in patients with epithelial ovarian cancer and their relationship to treatment with paclitaxel. Int J Gynecol Cancer.

[25] Qu QX, Zhu YB, Huang Q, Shen Y, Xu J, Zhang XG (2013). Tumor associated macrophage (tam) derived il-6 induced re-localization of pd-l1 in ovarian cancer cell [C]// 第十三届全国肿瘤生物治疗学术会议论文集.

[26] Wang X, Sun W, Shen W, Xia M, Chen C, Xiang D, Ning B, Cui X, Li H, Li X (2016). Long non-coding rna dilc regulates liver cancer stem cells via il-6/stat3 axis. J Hepatol.

[27] Zou A, Liu R, Wu X (2016). Long non-coding rna malat1 is up-regulated in ovarian cancer tissue and promotes sk-ov-3 cell proliferation and invasion. Neoplasma..

[28] Cheng Y, Li H, Deng Y, Tai Y, Zeng K, Zhang Y, Liu W, Zhang Q, Yang Y (2018). Cancer-associated fibroblasts induce pdl1+ neutrophils through the il6-stat3 pathway that foster immune suppression in hepatocellular carcinoma. Cell Death Dis.

[29] Wang L, Yi T, Kortylewski M, Pardoll DM, Zeng D, Yu H (2009). Il-17 can promote tumor growth through an il-6-stat3 signaling pathway. J Exp Med.

[30] Mace TA, Shakya R, Pitarresi JR, Swanson B, McQuinn CW, Loftus S, Nordquist E, Cruz-Monserrate Z, Yu L, Young G (2018). Il-6 and pd-l1 antibody blockade combination therapy reduces tumour progression in murine models of pancreatic cancer. Gut..

[31] Herbst RS, Soria JC, Kowanetz M, Fine GD, Hamid O, Gordon MS, Sosman JA, McDermott DF, Powderly JD, Gettinger SN (2014). Predictive correlates of response to the anti-pd-l1 antibody mpdl3280a in cancer patients. Nature..

[32] Wu F, Yin Z, Yang L, Fan J, Xu J, Jin Y, Yu J, Zhang D, Yang G (2019). Smoking induced extracellular vesicles release and their distinct properties in non-small cell lung cancer. J Cancer.

[33] Wang QM, Lian GY, Song Y, Huang YF, Gong Y (2019). Lncrna malat1 promotes tumorigenesis and immune escape of diffuse large b cell lymphoma by sponging mir-195. Life Sci.

[34] Lee Y, Shin JH, Longmire M, Wang H, Kohrt HE, Chang HY, Sunwoo JB (2016). Cd44+ cells in head and neck squamous cell carcinoma suppress t-cell-mediated immunity by selective constitutive and inducible expression of pd-l1. Clin Cancer Res.

[35] Tang B, Yan X, Sheng X, Si L, Cui C, Kong Y, Mao L, Lian B, Bai X, Wang X (2019). Safety and clinical activity with an anti-pd-1 antibody js001 in advanced melanoma or urologic cancer patients. J Hematol Oncol.

[36] Tada Y, Togashi Y, Kotani D, Kuwata T, Sato E, Kawazoe A, Doi T, Wada H, Nishikawa H, Shitara K (2018). Targeting vegfr2 with ramucirumab strongly impacts effector/ activated regulatory t cells and cd8(+) t cells in the tumor microenvironment. J Immunother Cancer.

