# Correction to: Long non-coding RNA HOTTIP enhances IL-6 expression to potentiate immune escape of ovarian cancer cells by upregulating the expression of PD-L1 in neutrophils

**DOI:** 10.1186/s13046-020-01755-z

**Published:** 2020-12-04

**Authors:** Anquan Shang, Weiwei Wang, Chenzheng Gu, Chen Chen, Bingjie Zeng, Yibao Yang, Ping Ji, Junjun Sun, Junlu Wu, Wenying Lu, Zujun Sun, Dong Li

**Affiliations:** 1 Department of Laboratory Medicine, Tongji Hospital of Tongji University School of Medicine, 389 Xincun Road, Shanghai, 200065 China; 2 Department of Pathology, The Sixth People’s Hospital of Yancheng City, Yancheng, 224001 China

**Correction to: J Exp Clin Cancer Res 38, 411 (2019)**

**https://doi.org/10.1186/s13046-019-1394-6**

Following publication of the original article [1], the journal was notified of discrepancies in the study dates listed in the ‘Study subjects’ section, as well as the methodology described throughout the ‘Materials and methods’ section. The authors were asked to address the concerns and have presented clarifications over the affected areas. In addition, the journal asked Tongji University School of Medicine to confirm the clarifications provided by the authors; the committee appointed by Tongji University School of Medicine confirmed that the corrected dates and methodology reflect the details of the performed study. The corrected areas can be found below. The authors declare that these corrections do not change the results or conclusions of this article and apologize for any confusion that may have been caused.

In the ‘Study subjects’ section (Page 2), the first sentence reads: “A total of 53 cases of OC tissues were collected from patients with OC who underwent surgical resection at Tongji Hospital of Tongji University School of Medicine and The Sixth People’s Hospital of Yencheng City from March 2017 to October 2018”; this should instead read: “A total of 53 cases of OC tissues were collected from patients with OC who underwent surgical resection at Tongji Hospital of Tongji University and The Sixth People’s Hospital of Yencheng City from March 2012 to October 2013, and follow-up was performed from May 2017 to October 2018.”

In the ‘Neutrophil isolation and identification’ section (Page 3), the third and fourth sentence reads: “The serum was diluted with aseptic PBS at the ratio of 1:1. Afterwards, 3 mL of lymphocyte separation medium was added into a 15-mL centrifuge tube, followed by careful addition into the diluted serum.” This should instead read: “The blood was diluted with aseptic PBS at the ratio of 1:1 after plasma removal. Afterwards, 3 mL of neutrophil separation medium Reagent A (Solarbio, Beijing, China) was added into a 15-mL centrifuge tube, and 2 ml of the PBS-diluted blood was carefully laid on top of the separation solution. An interface formed between these two layers of reagents due to their low mutual solubility and difference in density”

In the ‘Neutrophil isolation and identification’ section (Page 3), the sixth sentence reads: “The intermediate layer of peripheral blood mononuclear cells (PBMCs) was transferred to a new centrifuge tube, washed twice with PBS and then centrifuged at 1500 rpm for 10 min.” This should instead read: “Two circular milky layers of leukocytes formed after centrifugation. The upper layer are peripheral blood mononuclear cells, and the lower are neutrophil cells (Appendix). The supernatant and peripheral blood mononuclear cells layer were discarded. Carefully pipette the neutrophils layered between the red blood cell and reagent A into a new 15 ML centrifuge tube, and then wash with 10 ml PBS followed by centrifuge at 1500 rpm for 3 min. The yielded supernatant was discarded. Next, the tube was added with 5 ml of RBC lysis buffer, and then the cells were resuspended and lysed at room temperature for 10 min, and then centrifuged at 1500 rpm for 10 min and washed with PBS. The above steps were repeated once.” The authors have provided the Supplemental Figure 1 to accompany the above description of the Materials and Methods used.

## Figures and Tables

**Figure 1:**
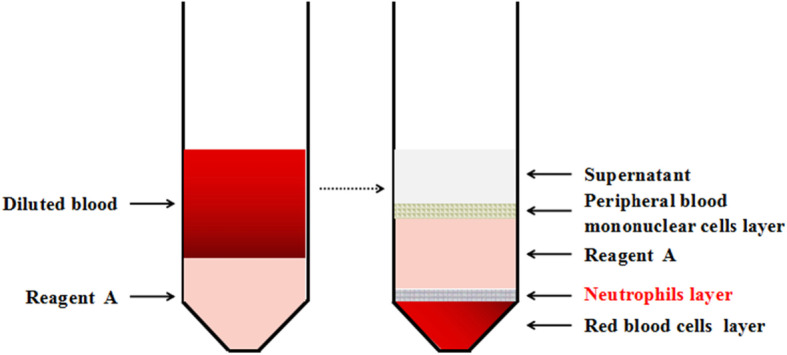
Schematic diagram of Neutrophil separation
